# Supplementary material for: DNA methylation landscapes in human cells and their chromatin determinants
Source: Ageing Cancer Res Treat. Author manuscript; Available in PMC 2026 Feb 4. (PMC12865891; doi:10.70401/acrt.2025.0007)
Supplement: Supplementary material. [file NIHMS2131420-supplement-Supplementary_material_.pdf]

Ageing and Cancer Research & Treatment
https://doi.org/10.70401/acrt.2025.0007

Supplementary information

DNA methylation landscapes in human cells and their chromatin determinants

Wei Cui, Zhijun Huang, Gerd P. Pfeifer

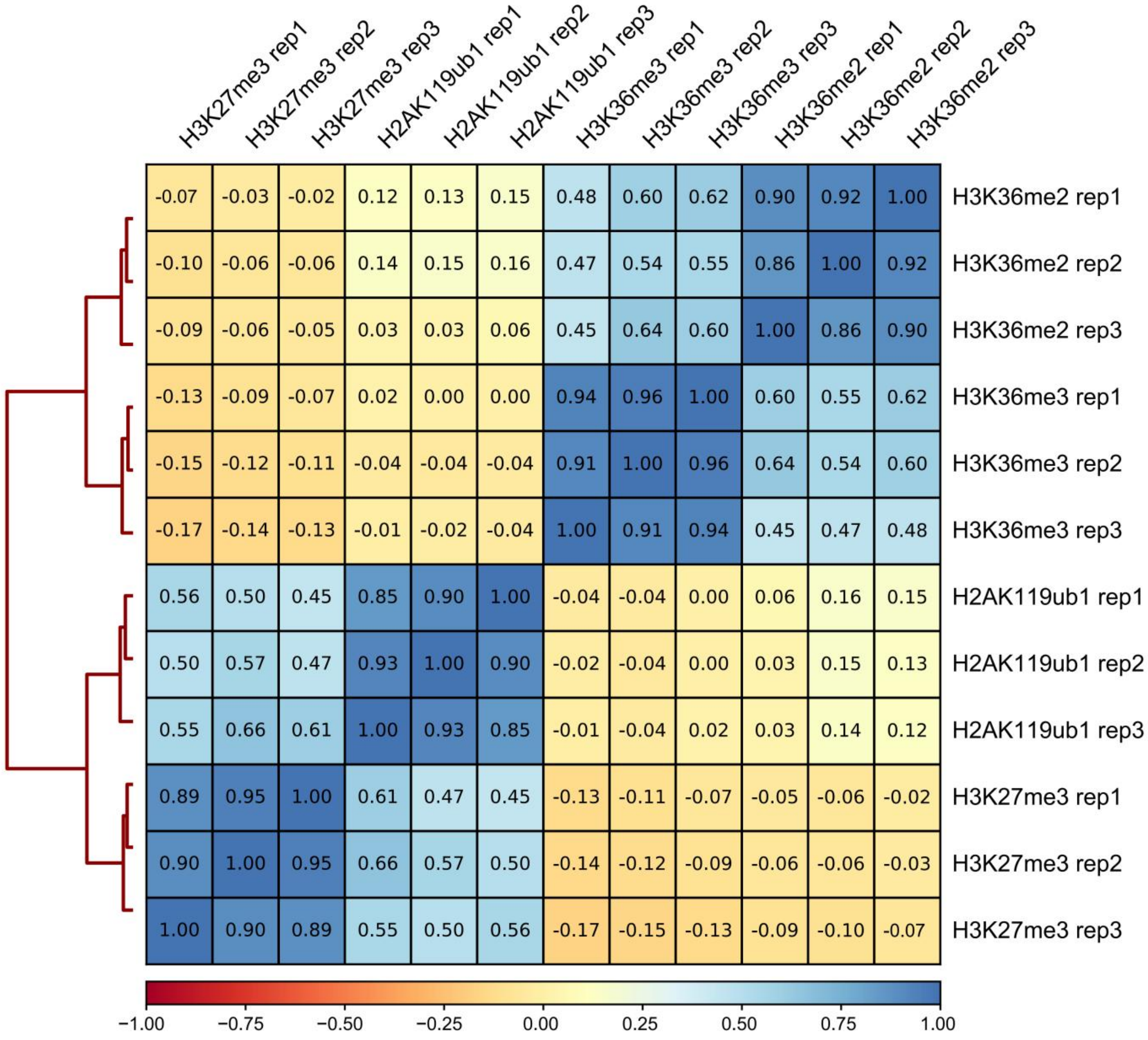

Figure S1. Genome-wide correlation of histone modifications within each group. The heatmap shows the Pearson's correlation coefficient for each histone modification within the three biological replicates.
